# Robust Self-Supported SnO_2_-Mn_2_O_3_@CC Electrode for Efficient Electrochemical Degradation of Cationic Blue X-GRRL Dye

**DOI:** 10.3390/molecules28093957

**Published:** 2023-05-08

**Authors:** Caiyun Li, Peng Yi, Junwei Sun, Xi-Ao Wang, Rongzhan Liu, Jiankun Sun

**Affiliations:** 1 College of Textiles and Clothing, Qingdao University, Qingdao 266071, China; 2 College of Chemistry and Chemical Engineering, Qingdao University, Qingdao 266071, China; 3 Collaborative Innovation Center for Eco-Textiles of Shandong Province and the Ministry of Education, 308 Ningxia Road, Qingdao 266071, China

**Keywords:** electrocatalytic degradation, SnO_2_-Mn_2_O_3_@CC electrode, cationic blue X-GRRL, recyclability

## Abstract

Exploration of highly efficient and robust catalyst is pivotal for electrocatalytic degradation of dye wastewater, but it still is a challenge. Here, we develop a three-dimensional self-supported SnO_2_-Mn_2_O_3_ hybrid nanosheets grown on carbon cloth (noted by SnO_2_-Mn_2_O_3_@CC) electrode via a simple hydrothermal method and annealing treatment. Benefitting from the interlaced nanosheets architecture that enlarges the surface area and the synergetic component effect that accelerates the interfacial electronic transfer, SnO_2_-Mn_2_O_3_@CC electrode exhibits a superior electrocatalytic degradation efficiency for cationic blue X-GRRL dye in comparison with the single metal oxide electrode containing SnO_2_@CC and Mn_2_O_3_@CC. The degradation efficiency of cationic blue X-GRRL on SnO_2_-Mn_2_O_3_@CC electrode can reach up to 97.55% within 50 min. Furthermore, self-supported architecture of nanosheets on carbon cloth framework contributes to a robust stability compared with the traditional electrode via the multiple dip/brush coating accompanied by the thermal decomposition method. SnO_2_-Mn_2_O_3_@CC electrode exhibits excellent recyclability, which can still retain a degradation efficiency of 94.12% after six cycles. This work may provide a new pathway for the design and exploration of highly efficient and robust electrooxidation catalysts for dye degradation.

## 1. Introduction

Water contamination has been a concerning social issue and has garnered extensive attention. Organic wastewater, originating from the massive discharge of chemical production, usually cause serious water pollution due to low biodegradability and complex composition [1,2,3]. Especially, dye contaminations and their intermediates constructed by aromatic group produced after degradation is toxic and carcinogenic, which seriously endangers aquatic life and human health [4,5,6].

Currently, the common methods to treat dye wastewater contains biodegradation, photocatalytic oxidation, adsorption method, flocculant method and membrane separation technology and so on [7,8,9,10,11,12,13,14]. Among them, electrochemical oxidation technology has been considered as an environment-friendly and promising candidate to treat dye wastewater because of its strong oxidation capacity, lack of secondary pollution effects, its reusability and easy operative qualities [15,16]. Electrochemical oxidation method mainly takes hydroxide radical (OH) or active chlorine species to degrade refractory and non-biodegradable organic contaminations. Additionally, the efficiency of electrochemical oxidation technology is greatly determined by anode materials [17].

To date, various anode materials, such as precious metal electrode materials (Pt), boron-doped diamond (BDD) and metal oxide electrode materials (SnO_2_, PbO_2_, RuO_2_, and IrO_2_), have been widely studied to oxidize organic dye for further degradation via electrochemical oxidation treatment [18,19]. Among them, SnO_2_ exhibits a potential advantage owing to its high oxygen evolution potential (OEP) and corrosion resistance. Generally, a high OEP can accelerate the oxidation process of organic dye pollution through the inhibition of oxygen evolution side-reaction. Nevertheless, SnO_2_ electrode has low conductivity and stability, which impedes the large-scale application of degrading dyes wastewater [20]. Therefore, exploiting high-performance anodes with large OEP, fast electronic transfer and high electrochemical oxidation stability is pivotal to efficient dye treatment [17]. Recent researches indicate that introducing foreign elements is a feasible method to enhance catalytic degradation performance of SnO_2_-based electrode [21]. Several introduced atoms include Sb, Ru, Ce, Nd and other rare earth elements [22,23,24,25,26,27,28]. For instance, Man et al. designed Sb and Ce co-doped SnO_2_ nanoflowers electrode which can boost the decolorization efficiency of organic pollutants due to a reduced charge transfer resistance induced by Ce-doped SnO_2_ [29]. Metal oxide are also used to improve the electrooxidation activity. Luu et al. fabricated Ti/SnO_2_-Nb_2_O_5_ bimetallic oxide electrode through the sol–gel method [30]. The experimental illustrates that the right amount of Nb_2_O_5_ can enhance the electrochemical activity and organic pollutant degradation efficiency of the Ti/SnO_2_-Nb_2_O_5_ electrode. It is worth noting that Mn_2_O_3_ seems to be a good candidate for electrode material because Mn_2_O_3_, as a p-type semiconductor material with a bandgap ranging from 1.2 to 1.29 eV, has been demonstrated to possess a faster interfacial electronic transfer between electrode and electrolyte in comparison with other high bandgap metal oxide nanomaterials [31].

In addition, regulating architecture to enhance the active area of electrode has also been proven as an efficient way, which can expose more active sites and favor catalytic oxidation performance [32,33,34,35]. Huang et al. developed a novel microsphere-structured Ti/SnO_2_-Sb electrode with small grain size and nanosheets architecture, which possessed a significantly enhanced electrochemical active surface area and degradation efficiency [34]. Hu et al. reported a Pb-modified SnO_2_ microsphere electrode that also presented an increased surface area to promote electrooxidation ability [36]. In addition to the high electrochemical degradation properties, the tight adherence between active oxide layer and substrate is another critical factor. Because traditional electrodes are usually synthesized with multiple dip/brush coating accompanied by the thermal decomposition method, vast cracks are likely to appear, thus the instable active layer on electrode substrate can cause a sharply decline of electrode activity or even deactivation [37]. In view of this, it is particularly important to synthesize a catalytic material with high catalytic performance and great stability.

In this work, three-dimensional SnO_2_-Mn_2_O_3_ hybrid nanosheets grown on carbon cloth (noted by SnO_2_-Mn_2_O_3_@CC) is synthesized using a simple hydrothermal and annealing treatment. The interconnected carbon fiber skeleton provides an efficient electronic transfer pathway, and the self-supported nanosheets arrays aligned on carbon cloth also strengthen the binding adherence between the active layer and the electrode scaffold substrate. Interestinglt, Mn precursor source can regulate the formation of nanosheets, which contributes to an increased surface area. Meanwhile, the synergistic effect of SnO_2_ and Mn_2_O_3_ can significantly reduce the interfacial electronic resistance of the electrode. As a result, SnO_2_-Mn_2_O_3_@CC electrode presents a higher OEP and faster interfacial electronic transfer. At 15 mA cm^−2^, the degradation rate of cationic blue X-GRRL dye based on SnO_2_-Mn_2_O_3_@CC electrode can reach up to 97.55% within 50 min. Moreover, SnO_2_-Mn_2_O_3_@CC electrode exhibits a good recyclability, which can still maintain stability with a degradation rate of 94.12% after six cycles.

## 2. Results and Discussion

### 2.1. Characterization

**Figure 1:**
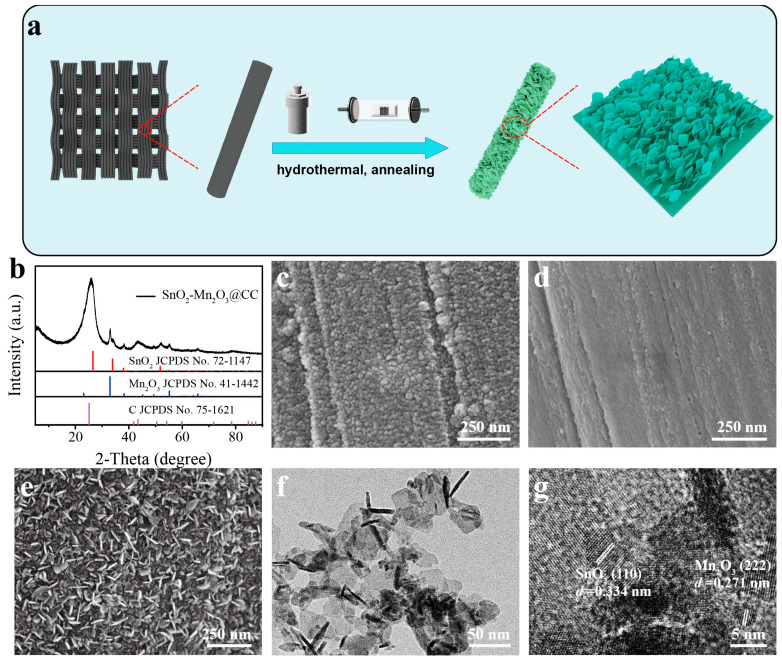
(**a**) Schematic illustration for the preparation process of SnO_2_-Mn_2_O_3_@CC. (**b**) XRD pattern of SnO_2_-Mn_2_O_3_@CC sample. (**c**–**e**) SEM images of Mn_2_O_3_@CC (**c**), SnO_2_@CC (**d**) and SnO_2_-Mn_2_O_3_@CC (**e**). TEM (**f**) and HRTEM (**g**) image of SnO_2_-Mn_2_O_3_ nanosheets.

The synthesis process of SnO_2_-Mn_2_O_3_@CC nanosheets grown on carbon cloth (CC) is shown in Figure 1a, wherein self-supported SnO_2_-Mn_2_O_3_@CC was synthesized using a simple hydrothermal reaction, followed by an annealing treatment at 400 °C. The crystal structure of these electrode materials was characterized using X-ray diffraction (XRD). In Figure 1b, the diffraction peaks at 26.2° and 44.2° are corresponded to the (002) and (101) crystal planes of C substrate (JCPDS No. 75-1621), respectively. The diffraction peaks located at 32.9°, 38.2°, 45.2°, 49.3° and 55.2° can be, respectively indexed to the (222), (400), (332), (431) and (440) crystal planes of Mn_2_O_3_ (JCPDS No. 41-1442). The residual diffraction peaks at 26.6°, 33.9°, 37.9° and 51.8° match well with the (110), (101), (200) and (211) crystal planes of SnO_2_ (JCPDS No. 72-1147), respectively. The above results confirm that the prepared sample is composed of SnO_2_, Mn_2_O_3_ and carbon substrate. Additionally, XRD pattern of the precursor in Figure S1 only detects the existence of SnO_2_ and MnF_2_, which further confirm the absence of Sn-MnO_x_ compound in the one-step preparation of SnO_2_-Mn_2_O_3_/CC electrode materials using the hydrothermal method. For comparison, the similar hydrothermal reaction and annealing process are repeated based on the single Sn or Mn source as precursor; the corresponding samples are, respectively denoted by SnO_2_@CC and Mn_2_O_3_@CC. XRD patterns in Figure S2 verify the successful preparation of SnO_2_@CC (Figure S2a) and Mn_2_O_3_@CC (Figure S2b) samples.

SEM images in Figure 1c,d and Figure S3 show that both Mn_2_O_3_@CC and SnO_2_@CC are composed of small and compact particles, while the surface of SnO_2_@CC sample presents a more tight-packed particles and seems to have a dense cover layer, which is verified by a broken surface with an intentional scratch (Figure S4). It is well known that a compact surface can block the contact between electrode and electrolyte, unavailing electrocatalytic activity. In contrast, the synthesized SnO_2_-Mn_2_O_3_@CC sample (Figure 1e) exhibits an obviously different morphology, which is composed of nanosheets with a thickness of 5 nm. As depicted in Figure 1e, the interlaced nanosheets are vertically aligned on the carbon fiber with irregular orientation. Compared with the tight-packed particles, the nanosheets-like morphology of SnO_2_-Mn_2_O_3_@CC sample offers an enhanced surface area, availing the connectivity of catalytic active sites on electrode and electrolyte. It is worth noting that the self-supported nanosheets on CC substrate show a robust stability in comparison with the common electrode via the multiple dip/brush coating accompanied by the thermal decomposition method. A hydrothermal and annealing treatment can promote the adhesion between active materials and the substrate scaffold, and avoid the active materials peeling from the substrate, thus contributing to good stability. In addition, the self-supported nanosheets structure can ensure a rapid electron transfer between the electrode and the active layer, which shortens the charge transfer pathway compared with the aggregated particles.

Transmission electron microscopy (TEM) and high-resolution transmission electron microscopy (HRTEM) were use to investigate the morphology and structure of SnO_2_-Mn_2_O_3_ nanosheets, which was prepared using the scratching from the carbon cloth framework. As shown in Figure 1f, SnO_2_-Mn_2_O_3_ presents a nanosheet morphology, consistent with SEM results. HRTEM image displays that the SnO_2_-Mn_2_O_3_ nanosheets are composed of crystalline nanoparticles (Figure 1g). The lattice fringes with a distance of 0.334 and 0.271 nm are corresponded with (110) plane of SnO_2_ and (222) plane of Mn_2_O_3_, respectively, which corroborates the fact that the nanosheet is composed of SnO_2_ and Mn_2_O_3_. The scanning TEM (STEM) image and energy dispersive X-ray spectroscopy (EDS) elemental mapping images (Figure S5) confirms the existence and homogeneous distribution of Sn, Mn and O elements on SnO_2_-Mn_2_O_3_ hybrid nanosheets. Additionally, the content of Mn_2_O_3_ is 57.8 wt% in the mixed oxide nanosheets (Figure S6 and Table S1). Based on the above result, we can deduce that the additive of Mn precursor source can not only regulate the morphology to form three-dimensional intersected nanosheets, but also promote the hybrid Mn_2_O_3_ and SnO_2_ composition.

**Figure 2:**
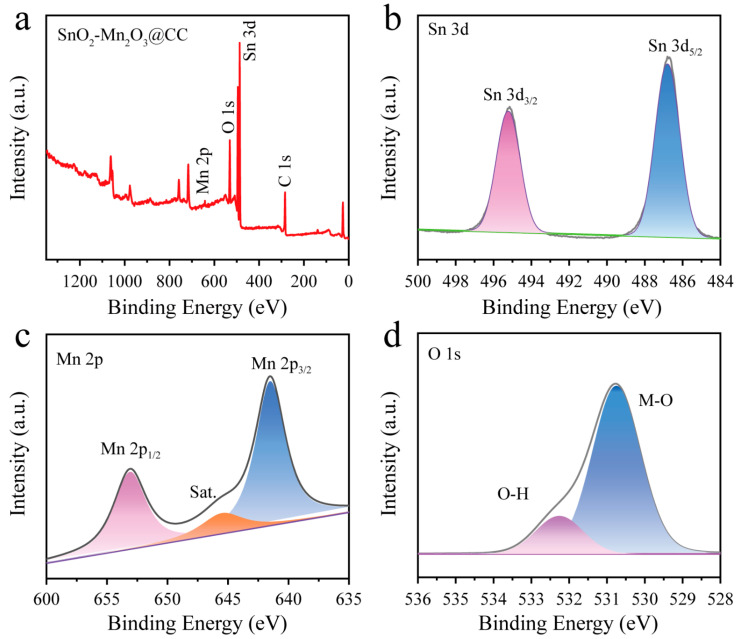
(**a**) Survey XPS spectrum and (**b**–**d**) high-resolution XPS spectra of Sn 3d (**b**), Mn 2p (**c**) and O 1s (**d**) for the SnO_2_-Mn_2_O_3_@CC sample.

X-ray photoelectron spectroscopy (XPS) measurement was performed to analyze the chemical composition and the valence state of the SnO_2_-Mn_2_O_3_@CC sample. In Figure 2a, the characteristic peaks of Sn, C, Mn and O can clearly be observed in the XPS survey, confirming the existence of Sn, C, Mn and O in SnO_2_-Mn_2_O_3_@CC. In Sn 3d XPS spectrum (Figure 2b), the peaks located at 487.2 and 495.6 eV are ascribed to Sn 3d_5/2_ and Sn 3d_3/2_, respectively. Additionally, binding energy difference between Sn 3d_3/2_ and Sn 3d_5/2_ is about 8.4 eV, indicating that Sn^4+^ exists in SnO_2_-Mn_2_O_3_@CC [29]. The Mn 2p XPS spectrum can be deconvoluted into three characteristic peaks (Figure 2c). The peaks at 641.5 and 653.1 eV, respectively correspond to Mn 2p_3/2_ and Mn 2p_1/2_, suggesting the existence of Mn^3+^ in Mn_2_O_3_, and the peak at 645.5 eV is the satellite peak (marked by Sat.). The O 1s spectrum (Figure 2d) can be fitted into two characteristic peaks. The O 1s peak at 530.75 eV is associated with the lattice oxygen (O_L_) existing in the metal oxide crystal. Peaks located at 532.26 eV is attributed to the adsorbed oxygen (O_ad_). In general, O_ad_ can promote the generation of ·OH during the electrooxidation process, owing to the easy exchange between O_ad_ and oxygen adsorbed molecules on the catalytic layer [38]. The aforementioned results indicate that SnO_2_ and Mn_2_O_3_ successfully coexisted in the prepared SnO_2_-Mn_2_O_3_@CC sample.

### 2.2. Electrochemical Characterization

**Figure 3:**
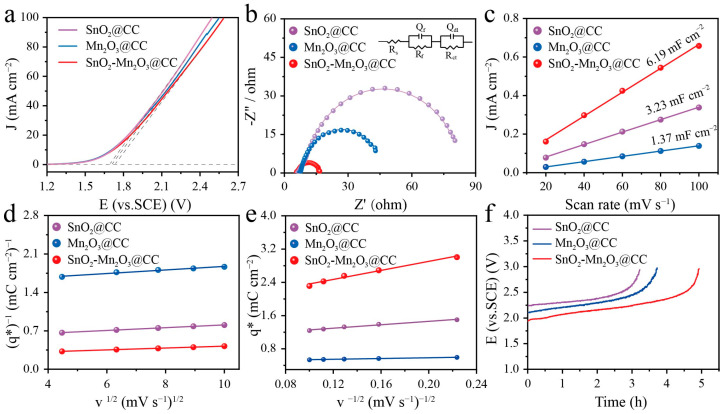
Electrochemical measurements of the SnO_2_@CC, Mn_2_O_3_@CC and SnO_2_-Mn_2_O_3_@CC samples in 0.5 M NaCl. (**a**) LSV curves. (**b**) Nyquist plots (inset is the equivalent circuit model diagram). (**c**) Current density vs. different scan rates and the corresponding linear slopes. (**d**) total voltammetric charge (q*_T_) plots and (**e**) outer voltammetric charge (q*_O_) plots, (**f**) accelerated lifetime curves in 0.5 M NaCl at 100 mA cm^−2^.

Linear voltammetry scanning (LSV) curves of different electrodes were tested in 0.5 M NaCl solution to evaluate the OEP of these electrodes, which can be obtained by the intersection value of the horizontal line and the tangent of LSV curve. OEP is an important factor to determine the electrochemical oxidation capacity of electrodes, because a large OEP value can restrain the oxygen evolution side reaction, thus providing more ·OH and larger current efficiency for electrooxidation reaction; meanwhile, it also restrains the oxidation of substrate produced by oxygen. Consequently, a larger OEP value usually suggests a superior electrooxidation catalytic activity of the electrode. As shown in Figure 3a, the OEP value of SnO_2_-Mn_2_O_3_@CC electrode is 1.73 V vs. the saturated calomel electrode (SCE), exceeding SnO_2_@CC (1.68 V vs. SCE) and Mn_2_O_3_@CC (1.70 V vs. SCE). These results suggest the SnO_2_-Mn_2_O_3_@CC electrode induced by Mn precursor can hinder the oxygen evolution side reaction on anode and contribute to a higher current efficiency in comparison with the single metal oxide electrode.

Interfacial impedance of different electrodes was evaluated using the electrochemical impedance spectroscopy (EIS). Nyquist plots and fitting results of different electrodes are displayed in Figure 3b and Table 1. In the equivalent circuit (the inset in Figure 3b), R_s_, R_f_ and R_ct_, respectively represent the solution resistance, oxide film resistance and charge transfer resistance on the interface of electrolyte and electrocatalyst; and Q_f_ and Q_dl_ depict the film capacitance and the double layer capacitance, respectively. R_ct_ of SnO_2_-Mn_2_O_3_@CC electrode is 8.51 Ω cm^2^, which is far less than SnO_2_@CC (70.65 Ω cm^2^) and Mn_2_O_3_@CC (34.53 Ω cm^2^). Moreover, the SnO_2_-Mn_2_O_3_ hybrid nanosheets (Table S2) also present the optimal intrinsic electrical conductivity. The aforementioned result verifies that the SnO_2_-Mn_2_O_3_ hybrid nanosheets induced by the Mn precursor additive can improve the charge transfer kinetics, which favors the electrochemical/electrooxidation catalytic activity of electrodes.

A large electrochemical active surface area (ECSA) is also an important factor in evaluating the catalytic activity area for a good electrode, which can afford more exposed catalytic active sites to promote electrocatalytic oxidation. According to ECSA = C_dl_/C_s_, ECSA is proportional to the double-layer capacitance (C_dl_), thus C_dl_ can be used to assess the ECSA and can be calculated using cyclic voltammetry (CV). In Figure S7, CV curves of different electrodes are tested in the non-Faraday region from 0.5 to 0.7 V vs. SCE in 0.5 M NaCl solution at a scanning rate ranging from 20 to 100 mV s^−1^. As shown in Figure 3c, SnO_2_-Mn_2_O_3_@CC exhibits the largest C_dl_ value (6.19 mF cm^−2^), exceeding SnO_2_@CC (3.23 mF cm^−2^) and Mn_2_O_3_@CC (1.37 mF cm^−2^). The result suggests that hybrid SnO_2_-Mn_2_O_3_@CC can afford the largest catalytic surface area, matched well with SEM results.

Voltammetric charge (q*) is also a critical parameter to assess the active surface area of electrodes, which is relevant to the specific electroactivity of the sites and can be obtained via CV curves [39]. It is worth noting that the total voltammetric charge (q*_T_) contains the outer voltammetric charge (q*_O_) and the inner voltammetric charge (q*_I_), which can be calculated from the relationship of q* and scan rate (*v*). The detailed equations are as follows:

(1) $$q^{*}{=q}_{O}^{*}{\;+\;k}_{1}v^{-1/2}$$

(2) $${{(q}^{*})}^{-1}{=(q}_{T}^{*}{)}^{-1}{\;+\;k}_{2}v^{1/2}$$

(3) $$q_{T}^{*}\;{=q}_{O}^{*}{\;+\;q}_{I}^{*}$$

At a very low scan rate, the total active surface containing the inner and outer of electrode can participate in the reaction, thus the total voltammeric charge q*_O_ can be calculated by Equation (1). The intercept of the straight line in Figure 3d is the reciprocal of q*_T_, whereas at a high scan rate, especially when *v* approaches to ∞, electrolyte ions only contact with the outer surface of electrode, and has no time to permeate into the inside of the electrode, thus only q*_O_ contributes to the charge. According to Equation (2), q*_O_ is equal to the intercept of q* vs. *v*^−1/2^ (Figure 3e). As listed in Table 2, SnO_2_-Mn_2_O_3_@CC electrode presents the largest total voltammetric charge, which is, 2.2 and 6.2 times of SnO_2_@CC and Mn_2_O_3_@CC, respectively. Similarly, both q*_O_ and q*_I_ value of SnO_2_-Mn_2_O_3_@CC are still greatly larger than SnO_2_@CC and Mn_2_O_3_@CC electrodes. These above results further confirm that the active surface area of hybrid SnO_2_-Mn_2_O_3_@CC is larger than the single metal oxide electrode, which can ascribe to the three-dimensional nanosheets architecture produced by the addition of Mn precursor.

Service life of electrode is another pivotal factor to estimate the catalytic activity, which determines its further practical application. An accelerated life test of different electrode was carried out at 100 mA cm^−2^. As manifested in Figure 3f, SnO_2_-Mn_2_O_3_@CC electrode exhibits the longest service life, exceeding that of SnO_2_@CC and Mn_2_O_3_@CC, which indicates that the hybrid SnO_2_-Mn_2_O_3_@CC electrode induced by the addition of Mn precursor source can improve electrode service life and stability. Interestingly, the service life of these electrodes is superior than the traditional dip/brush-coating electrodes [40], which may be ascribed to the tight adhesion between catalytic active layer and substrate generated using the hydrothermal and annealing process. Therefore, the self-supported structure on substrate seems as a good candidate for a robust electrooxidation catalyst.

### 2.3. Electrochemical Degradation of Cationic Blue X-GRRL

**Figure 4:**
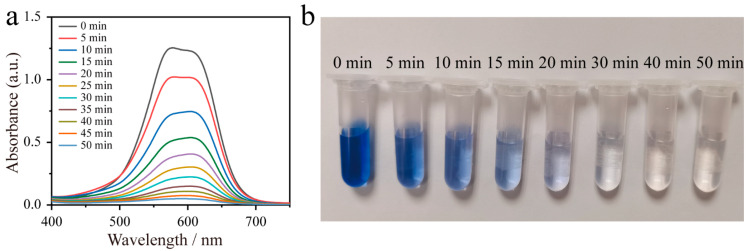
(**a**) UV–vis absorbance spectra and (**b**) time-dependent photos of the solutions in the degradation process (initial concentration of cationic blue X-GRRL is 20 mg L^−1^; supporting electrolyte is 0.5 M NaCl, current density is 15 mA cm^−2^).

Electrooxidation ability of these prepared electrode materials were evaluated based on the degradation of the cationic blue X-GRRL dye. The time-dependent Ultraviolet Spectrophotometer (UV–vis) absorbance spectra of cationic blue X-GRRL on SnO_2_-Mn_2_O_3_@CC electrode are shown in Figure 4a, which is a pivotal factor to estimate the degradation efficiency of cationic blue X-GRRL. The azo conjugated chromogenic system in cationic blue X-GRRL dye molecule corresponds to the absorption peak of 608 nm [41]. In Figure 4a, the intensity of characteristic adsorption peak at 608 nm decrease with time and disappears after 50 min, indicating that cationic blue X-GRRL has been completely decomposed in 50 min. Meanwhile, a visually gradual color fading of cationic blue X-GRRL dye is depicted in the time-dependent photos (Figure 4b). The color is near to colorless at 40 min, suggesting that cationic blue X-GRRL dye has been successfully degraded in the electrochemical oxidation process. Moreover, the time-dependent UV–vis absorbance spectra of cationic blue X-GRRL on SnO_2_@CC and Mn_2_O_3_@CC electrodes are also tested (Figure S8a,b). The comparative intensity of time-dependent characteristic adsorption peaks at 608 nm (Figure S8c) indicate a faster degradation process of cationic blue X-GRRL on SnO_2_-Mn_2_O_3_@CC electrode.

**Figure 5:**
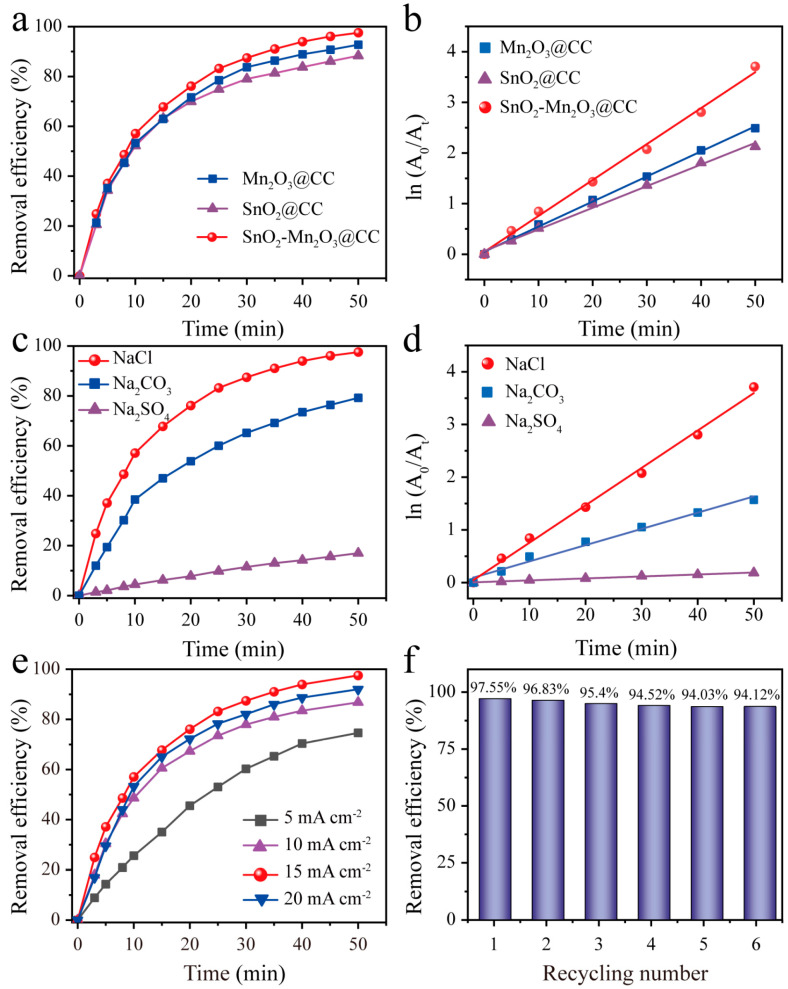
(**a**,**b**) Degradation efficiency (**a**) and kinetic fitting (**b**) of cationic blue X-GRRL dye on different electrodes. (**c**,**d**) Degradation efficiency (**c**) and kinetic fitting (**d**) of cationic blue X-GRRL dye on SnO_2_-Mn_2_O_3_@CC electrode in different supporting electrolyte. (**e**) Degradation efficiency of cationic blue X-GRRL dye at different current density. (**f**) Recyclability of SnO_2_-Mn_2_O_3_@CC electrode for degradation of cationic blue X-GRRL.

The electrocatalytic oxidation performances of different electrodes for degrading cationic blue X-GRRL with an initial concentration of 20 mg L^−1^ were investigated. In Figure 5a, after 50 min of electrolysis, the removal efficiency of cationic blue X-GRRL on SnO_2_-Mn_2_O_3_@CC is 97.55%, which is superior than that of Mn_2_O_3_@CC (92.74%) and SnO_2_@CC electrode (88.3%). Meanwhile, the relationship between time and electrochemical degradation of cationic blue X-GRRL on different electrodes follows the pseudo-first-order kinetics model, which can be described as the following equation:

(4) $${-\ln(A}_{t}{/A}_{0})\;=\;kt$$

where, A_0_ is the initial absorbance, A_t_ is the absorbance at given time t and *k* is the kinetic rate constant. As displayed in Figure 5b, the reaction kinetic rate constant *k*_1_ of SnO_2_-Mn_2_O_3_@CC is larger than Mn_2_O_3_@CC and SnO_2_@CC electrodes, indicating a faster cationic blue X-GRRL degradation rate. The detailed values of k on different electrodes are listed in Table S3. Different kinds of supporting electrolyte have varied influences on the electrocatalytic degradation efficiency. Therefore, different electrolytes containing NaCl, Na_2_SO_4_ and Na_2_CO_3_ were used to investigate the effect of electrolyte ions on degrading cationic blue X-GRRL. As displayed in Figure 5c, the degradation efficiency of cationic blue X-GRRL on SnO_2_-Mn_2_O_3_@CC electrode in three electrolytes increase along with time. Additionally, the dye degradation efficiency in 0.5 M NaCl electrolyte can be as high as 97.55% after 50 min, which greatly exceeds that of Na_2_SO_4_ (17%) and Na_2_CO_3_ (79.23%). During the electrooxidation process, HO· free radicals are responsible for the dye degradation, which is verified by the time-dependent fluorescence spectrometry (Figure S9), moreover, chlorine species also plays an important role. Since the redox potential of Cl^−^/Cl_2_ (U = 1.36 V vs. RHE) is low than that of HO·/H_2_O (U = 2.2 V vs. RHE) [42], Cl_2_ is more easily generated in comparison with HO· in wastewater, along with a series of the following reactions [43]:

(5) $${\mathrm{Cl}}^{-}\to {\;\mathrm{Cl}+e}^{-}$$

(6) $$2\cdot \mathrm{Cl}\to {\;\mathrm{Cl}}_{2}$$

(7) $${\mathrm{Cl}}_{2}{+H}_{2}O\;\to {\;\mathrm{HClO}+H}^{+}{+\mathrm{Cl}}^{-}$$

(8) $$\mathrm{HClO}\;\to H^{+}{+\mathrm{ClO}}^{-}$$

(9) $$\mathrm{HClO}+\mathrm{organics}\to {\mathrm{CO}}_{2}{+H}_{2}O+\mathrm{HCl}$$

Therefore, SnO_2_-Mn_2_O_3_@CC electrode in 0.5 M NaCl electrolyte can contribute to the excellent electrocatalytic oxidation efficiency. Moreover, the degradation of cationic blue X-GRRL in different electrolytes follows the pseudo-first-order kinetics (Figure 5d). SnO_2_-Mn_2_O_3_@CC electrode displays the fastest reaction degrading rate in 0.5 M NaCl electrolyte, and the corresponding reaction rate constant is listed in Table S4.

Current density is another critical factor affecting the electrocatalytic degradation ability of electrodes. Figure 5e shows the degradation efficiency of cationic blue X-GRRL on SnO_2_-Mn_2_O_3_@CC electrode in 0.5 M NaCl electrolyte by applying 5, 10, 15 and 20 mA cm^−2^. The degradation efficiency of cationic blue X-GRRL shows a remarkable enhancement with the increased current density ranging from 5 to 15 mA cm^−2^, while the degradation efficiency decreased when the current density is 20 mA cm^−2^. An excessive current density along with a higher potential can impel the oxygen evolution side reaction, restraining electrochemical oxidation reaction. Moreover, excessive current density is a waste of electric energy and economic cost. Therefore, 15 mA cm^−2^ is an optimal condition for degrading cationic blue X-GRRL.

The recyclability of electrode is a pivotal issue in its practical application. SnO_2_-Mn_2_O_3_@CC electrode is used for recyclable degradation of cationic blue X-GRRL for six times. As shown in Figure 5f, the removal efficiency of cationic blue X-GRRL almost remains constant for six cycles. The successive degradation rates at the 50th min are 97.55%, 96.83%, 95.4%, 94.52%, 94.03%, and 94.12%, respectively. The above result confirms that SnO_2_-Mn_2_O_3_@CC electrode as an electrooxidation catalyst for cationic blue X-GRRL dye possesses a good recyclability.

**Figure 6:**
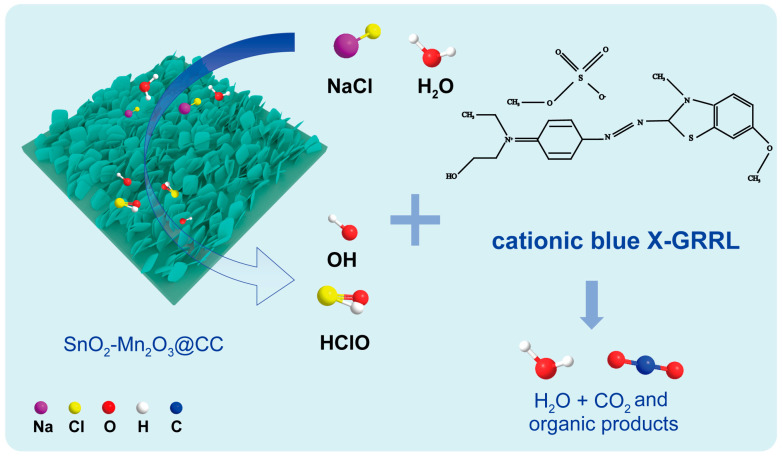
Electrocatalytic degradation mechanism of cationic blue X-GRRL on SnO_2_-Mn_2_O_3_@CC.

As shown in Figure 6, the electrocatalytic degradation mechanism of cationic blue X-GRRL on SnO_2_-Mn_2_O_3_@CC is proposed based on the aforementioned results in the degradation experiments. Firstly, H_2_O and Cl^−^ on the surface of SnO_2_-Mn_2_O_3_@CC electrode lose electrons to form hydroxyl radical (·OH) and active chlorine in the NaCl solution, and the active chlorine further hydrolyzes into ClO^−^. Then, the ·OH radical and ClO^−^ combine with cationic blue X-GRRL dye molecule and degrade the dye molecule to produce CO_2_ and H_2_O [41]. In the electrocatalytic degradation process of cationic blue X-GRRL on SnO_2_-Mn_2_O_3_@CC, active chlorine may be the main factor for the rapid degradation.

## 3. Materials and Methods

### 3.1. Materials

T Stannous chloride dihydrate (SnCl_2_⋅2H_2_O, 98.0%), manganese acetate tetrahydrate (MnC_4_H_6_O_4_⋅4H_2_O, 99.0%), ammonium fluoride (NH_4_F, 96.0%), sodium carbona anhydrous (Na_2_CO_3_, 99.8%), sodium chloride (NaCl, 99.5%), ethanol (C_2_H_5_OH, 99.7%) and sodium sulfate anhydrous (Na_2_SO_4_, 99.0%) were purchased from Sinopharm chemical Reagent Co, Ltd. (Shanghai, China). A commercial cationic blue X-GRRL was obtained from Jiangsu Jinji Industrial Co., Ltd. (China).

### 3.2. Preparation of SnO_2_-Mn_2_O_3_@CC

Firstly, the carbon cloth substrate was treated in HNO_3_ and rinsed with deionized water and ethanol. Typically, 2 mmol MnC_4_H_6_O_4_·4H_2_O, 2 mmol SnCl_2_·2H_2_O and 5 mmol of NH_4_F were dissolved in 20 mL of solvent containing ethanol and deionized water with a volume ratio of 1:1, then a carbon cloth (2 × 3 cm^2^) and the above solution was transferred to a 50 mL autoclave at 180 °C for 6 h. After washing it several times, the prepared sample was dried at 60 °C; for 8 h. Finally, the SnO_2_-Mn_2_O_3_@CC was synthesized by annealing at 400 °C; for 2 h in air atmosphere.

For SnO_2_ @CC and Mn_2_O_3_@CC, all the above processes were repeated, except for the initial precursor sources. SnO_2_ @CC contains 2 mmol SnCl_2_·2H_2_O and 5 mmol of NH_4_F, while Mn_2_O_3_@CC includes 2 mmol MnC_4_H_6_O_4_·4H_2_O and 5 mmol of NH_4_F.

### 3.3. Characterization Measurements

The crystalline structures of the electrodes were investigated using the X-ray diffraction (XRD) equipment (Rigaku D-MAX 2500/PC, Tokyo, Japan) with a Cu Ka radiation source (λ = 0.15405 nm). The surface morphology of the electrode materials was analyzed using scanning electron microscopy (SEM, Tescan MIRA4, Jebulno, Czech Republic) and transmission electron microscopy (TEM, Tecnai G2F30, Hillsboro, OR, USA). The chemical composition and oxidation state were detected on X-ray photoelectron spectrometer (XPS, Thermo Scientific, ESCALAB 250XI, Waltham, MA, USA) with a Mg-Kα radiation source. The degradation experiment was carried out on an ultraviolet spectrophotometer (UV–vis, Beijing Puxi General Instrument Co., Ltd., Beijing, China).

### 3.4. Electrochemical Testing

All of the electrochemical tests were carried out on a CHI 760D electrochemical workstation in a three-electrode system at 25 °C;. A total of 0.5 M NaCl solution was served as the electrolyte solution. The synthetic material (1 × 1 cm^2^) was served as the working electrode, and a platinum sheet and saturated calomel electrode (SCE) were served as the counter electrode and the reference electrode, respectively. Electrochemical impedance spectroscopy (EIS) curves were examined under 0.54 V vs. SCE at a frequency ranging from 10^−2^ to 10^−5^ Hz in 0.5 M NaCl. The double layer capacitance (C_dl_) of the material was implemented using cyclic voltammetry (CV) tested in the non-Faraday region from 0.5 to 0.7 V (vs. SCE). Accelerated service life tests of different electrodes were performed at 100 mA cm^−2^ in 0.5 M NaCl. Electrodes were regarded as devitalized when the applied potential exceeded 3 V.

### 3.5. Electrocatalytic Experiments

The electrooxidation degradation of cationic blue X-GRRL dye was carried out at 15 mA cm^−2^ in 100 mL solution containing cationic blue X-GRRL solution (20 mg L^−1^) and NaCl (0.5 M). During the degradation process, 2 mL of solution was taken out at a special interval and its concentration and absorbance were detected using the Ultraviolet Spectrophotometer (UV–vis). The removal efficiency of dye was described as follows:

(10) $$\mathrm{Removal}\;\mathrm{efficiency}\;\left(\%\right)=\frac{A_{0}{\;-\;A}_{t}}{A_{0}}\times \;100\%$$

where, A_0_ is the initial absorbance, and A_t_ is the absorbance at given time *t*.

Recyclability of SnO_2_-Mn_2_O_3_@CC electrode was performed at 15 mA cm^−2^. After every degradation cycle, SnO_2_-Mn_2_O_3_@CC electrode was washed with water for several times and was directly used for next degradation cycle.

## 4. Conclusions

In summary, self-supported SnO_2_-Mn_2_O_3_ hybrid nanosheets grown on carbon cloth is successfully synthesized using a simple hydrothermal and annealing treatment. The addition of Mn precursor can regulate the formation of nanosheets to the interlaced network architecture, which contributes to an increased surface area and more exposed active sites. Meanwhile, Mn precursor dopant can enrich the component of active layer, thus the synergistic effect of SnO_2_ and Mn_2_O_3_ accelerate a faster interfacial electronic transfer. Self-supported nanosheets on carbon cloth contribute to a robust stability compared with the traditional electrode via the multiple dip/brush coating accompanied by the thermal decomposition method. Consequently, SnO_2_-Mn_2_O_3_@CC electrode possesses a larger electrochemical active area and improved q*_T_, q*_O_ and q*_I_ value. The degradation efficiency of cationic blue X-GRRL on SnO_2_-Mn_2_O_3_@CC electrode can reach up to 97.55% within 50 min and the electrocatalytic oxidation process follows the first-order kinetic. Moreover, SnO_2_-Mn_2_O_3_@CC electrode exhibits excellent recyclability, which can still retain a degradation efficiency of 94.12% after six cycles. This excellent electrocatalytic oxidation activity indicates that the self-supported SnO_2_-Mn_2_O_3_@CC electrode is a promising electrooxidation catalyst for dye degradation.

## Figures and Tables

**Table 1:** The impedance fitting results for different electrodes.

	R_s_ (Ω cm^2^)	R_f_ (Ω cm^2^)	R_ct_ (Ω cm^2^)	Q_f_ (mF cm^2^)	Q_dl_ (mF cm^2^)
SnO_2_-Mn_2_O_3_@CC	5.887	1.629	8.509	1.32	2.27
SnO_2_@CC	6.577	4.051	70.65	0.21	0.52
Mn_2_O_3_@CC	7.329	1.604	34.53	0.91	0.79

**Table 2:** Electrochemical analysis results under different scan rates of the prepared anodes.

	q*_T_ (mC cm^−2^)	q*_O_ (mC cm^−2^)	q*_I_ (mC cm^−2^)
SnO_2_-Mn_2_O_3_@CC	3.991	1.879	2.112
SnO_2_@CC	1.7999	1.0491	0.7508
Mn_2_O_3_@CC	0.6456	0.49192	0.1537

## Data Availability

All data are available upon reasonable request from the corresponding author.

## Supplementary Materials

The following supporting information can be downloaded at: https://www.mdpi.com/article/10.3390/molecules28093957/s1, Figure S1: XRD pattern of SnO_2_-Mn_2_O_3_@CC precursor; Figure S2: XRD patterns of SnO_2_@CC (a) and Mn_2_O_3_@CC (b). Figure S3: SEM images of Mn_2_O_3_@CC (a) and SnO_2_@CC (b). Figure S4: SEM images of carbon cloth (a) and SnO_2_@CC (b). Figure S5: STEM image and corresponding EDS elemental mapping images of Sn, O, Mn. Figure S6: EDX spectrum of SnO_2_-Mn_2_O_3_@CC. Figure S7: Cyclic voltammograms of Mn_2_O_3_@CC (a), SnO_2_@CC (b), SnO_2_-Mn_2_O_3_@CC (c) at the different scan rates varying from 20 to 100 mV s^−1^ in 1M KOH Figure S8: UV-vis absorbance spectrum of (a) Mn_2_O_3_@CC electrode and (b) SnO_2_@CC electrode in the degradation process (initial concentration of cationic blue X-GRRL is 20 mg L^−1^; supporting electrolyte is 0.5 M NaCl, current density is 15 mA cm^−2^). (c) The comparative intensity of time-dependent characteristic adsorption peaks of cationic blue X-GRRL on different electrodes. Figure S9: Variations of fluorescence intensity at different time (p-phthalic acid: 0.5 mM, NaCl: 0.5 M). Table S1: The elemental content in SnO_2_-Mn_2_O_3_ hybrid nanosheets. Table S2: The electrical conductivity of different electrodes. Table S3: Kinetic parameters of different electrodes for degradation of cationic blue X-GRRL. Table S4: Kinetic parameters of SnO_2_-Mn_2_O_3_@CC electrode in different electrolytes for degradation of cationic blue X-GRRL.

## Sample Availability

Samples of the compounds are available from the authors.

## References

[1] Ma D., Yi H., Lai C., Liu X., Huo X., An Z., Li L., Fu Y., Li B., Zhang M. (2021). Critical review of advanced oxidation processes in organic wastewater treatment. Chemosphere.

[2] Uddin M.J., Ampiaw R.E., Lee W. (2021). Adsorptive removal of dyes from wastewater using a metal-organic framework: A review. Chemosphere.

[3] Zhang M.-H., Dong H., Zhao L., Wang D.-X., Meng D. (2019). A review on Fenton process for organic wastewater treatment based on optimization perspective. Sci. Total Environ..

[4] El Gaayda J., Titchou F.E., Barra I., Karmal I., Afanga H., Zazou H., Yap P.-S., Abidin Z.Z., Hamdani M., Akbour R.A. (2022). Optimization of turbidity and dye removal from synthetic wastewater using response surface methodology: Effectiveness of Moringa oleifera seed powder as a green coagulant. J. Environ. Chem. Eng..

[5] Shao Z., Jiang W., Meng H., Sui Y., Meng Y. (2023). Fabrication of the high efficient novel SiC foam based 3D metal oxide anodes with long life to improve electrocatalytic oxidation performance. J. Environ. Chem. Eng..

[6] Xia Y., Wang G., Guo L., Dai Q., Ma X. (2020). Electrochemical oxidation of Acid Orange 7 azo dye using a PbO(2) electrode: Parameter optimization, reaction mechanism and toxicity evaluation. Chemosphere.

[7] Kanagaraj T., Thiripuranthagan S. (2017). Photocatalytic activities of novel SrTiO_3_-BiOBr heterojunction catalysts towards the degradation of reactive dyes. Appl. Catal. B Environ..

[8] Yafang W., Lin Y., Rui Z., Jianwu L., Anrong Y., Hongyan X., Shaojian L. (2022). Z-scheme CeO_2_/Ag/CdS heterojunctions functionalized cotton fibers as highly recyclable and efficient visible light-driven photocatalysts for the degradation of dyes. J. Clean. Prod..

[9] Vasiraja N., Prabhahar R.S.S., Joshua A. (2023). Preparation and Physio–Chemical characterisation of activated carbon derived from prosopis juliflora stem for the removal of methylene blue dye and heavy metal containing textile industry effluent. J. Clean. Prod..

[10] Kim E.-J., Lee C.-S., Chang Y.-Y., Chang Y.-S. (2013). Hierarchically Structured Manganese Oxide-Coated Magnetic Nanocomposites for the Efficient Removal of Heavy Metal Ions from Aqueous Systems. Acs Appl. Mater. Interfaces.

[11] Rashad M.M., Ismail A.A., Osama I., Ibrahim I.A., Kandil A.H.T. (2014). Decomposition of Methylene Blue on Transition Metals Doped SnO_2_ Nanoparticles. CLEAN—Soil Air Water.

[12] Chen Y., Sun R., Yan W., Wu M., Zhou Y., Gao C. (2022). Antibacterial polyvinyl alcohol nanofiltration membrane incorporated with Cu(OH)_2_ nanowires for dye/salt wastewater treatment. Sci. Total Environ..

[13] Sadia M., Ahmad I., Ul-Saleheen Z., Zubair M., Zahoor M., Ullah R., Bari A., Zekker I. (2023). Synthesis and Characterization of MIPs for Selective Removal of Textile Dye Acid Black-234 from Wastewater Sample. Molecules.

[14] Hasan I., Albaeejan M.A., Alshayiqi A.A., Al-Nafaei W.S., Alharthi F.A. (2023). In Situ Hydrothermal Synthesis of Ni_1_-xMnxWO_4_ Nanoheterostructure for Enhanced Photodegradation of Methyl Orange. Molecules.

[15] He Z., Chen J., Chen Y., Makwarimba C.P., Huang X., Zhang S., Chen J., Song S. (2018). An activated carbon fiber-supported graphite carbon nitride for effective electro-Fenton process. Electrochim. Acta.

[16] Rodriguez-Narvaez O.M., Picos A.R., Bravo-Yumi N., Pacheco-Alvarez M., Martinez-Huitle C.A., Peralta-Hernandez J.M. (2021). Electrochemical oxidation technology to treat textile wastewaters. Curr. Opin. Electrochem..

[17] Wang G., Zhang H., Wang W., Zhang X., Zuo Y., Tang Y., Zhao X. (2021). Fabrication of Fe-TiO_2_-NTs/SnO_2_-Sb-Ce electrode for electrochemical degradation of aniline. Sep. Purif. Technol..

[18] Zhuo Q., Wang J., Niu J., Yang B., Yang Y. (2020). Electrochemical oxidation of perfluorooctane sulfonate (PFOS) substitute by modified boron doped diamond (BDD) anodes. Chem. Eng. J..

[19] Rodrigues de Oliveira G., Suely Fernandes N., Vieira de Meloa J., Ribeiro da Silva D., Urgeghe C., Martinez-Huitle C.A. (2011). Electrocatalytic properties of Ti-supported Pt for decolorizing and removing dye from synthetic textile wastewaters. Chem. Eng. J..

[20] Santos G.d.O.S., Vasconcelos V.M., da Silva R.S., Rodrigo M.A., Eguiluz K.I.B., Salazar-Banda G.R. (2020). New laser-based method for the synthesis of stable and active Ti/SnO_2_-Sb anodes. Electrochim. Acta.

[21] Sun Y., Cheng S., Yu Z., Li L., Li C., Yang J. (2020). Elucidating deactivation mechanisms of Pd-doped and un-doped Ti/SnO_2_-Sb electrodes. J. Alloy. Compd..

[22] Ahn Y.Y., Yang S.Y., Choi C., Choi W., Kim S., Park H. (2017). Electrocatalytic activities of Sb-SnO_2_ and Bi-TiO_2_ anodes for water treatment: Effects of electrocatalyst composition and electrolyte. Catal. Today.

[23] Kong J.-T., Shi S.-Y., Zhu X.-P., Ni J.-R. (2007). Effect of Sb dopant amount on the structure and electrocatalytic capability of Ti/Sb-SnO_2_ electrodes in the oxidation of 4-chlorophenol. J. Environ. Sci..

[24] Watts R.J., Wyeth M.S., Finn D.D., Teel A.L. (2008). Optimization of Ti/SnO_2_-Sb_2_O_5_ anode preparation for electrochemical oxidation of organic contaminants in water and wastewater. J. Appl. Electrochem..

[25] Chen S., Zhou L., Yang T., He Q., Zhou P., He P., Dong F., Zhang H., Jia B. (2020). Thermal decomposition based fabrication of dimensionally stable Ti/SnO(2)-RuO(2) anode for highly efficient electrocatalytic degradation of alizarin cyanin green. Chemosphere.

[26] Yang K., Liu Y., Qiao J. (2017). Electrodeposition preparation of Ce-doped Ti/SnO_2_-Sb electrodes by using selected addition agents for efficient electrocatalytic oxidation of methylene blue in water. Sep. Purif. Technol..

[27] Chen A., Xia S., Pan H., Xi J., Qin H., Lu H., Ji Z. (2018). A promising Ti/SnO_2_ anodes modified by Nb/Sb co-doping. J. Electroanal. Chem..

[28] Sun Z., Zhang H., Wei X., Du R., Hu X. (2015). Fabrication and Electrochemical Properties of a SnO_2_-Sb Anode Doped with Ni-Nd for Phenol Oxidation. J. Electrochem. Soc..

[29] Man S., Zeng X., Yin Z., Yang H., Bao H., Xu K., Wang L., Ge X., Mo Z., Yang W. (2022). Preparation of a novel Ce and Sb co-doped SnO_2_ nanoflowers electrode by a two-step (hydrothermal and thermal decomposition) method for organic pollutants electrochemical degradation. Electrochim. Acta.

[30] Le Luu T., Ngan P.T.K. (2023). Fabrication of high performance Ti/SnO_2_-Nb_2_O_5_ electrodes for electrochemical textile wastewater treatment. Sci. Total Environ..

[31] Koventhan C., Vinothkumar V., Chen S.-M. (2022). Rational design of manganese oxide/tin oxide hybrid nanocomposite based electrochemical sensor for detection of prochlorperazine (Antipsychotic drug). Microchem. J..

[32] Li G., Li G., Wang H., Xiang C., Zhuang J., Liu Q., Tang H. (2015). Preparation of Sb Doped Nano SnO_2_/Porous Ti Electrode and Its Degradation of Methylene Orange. Rare Met. Mater. Eng..

[33] Xu L., Lian Y. (2016). A Ti/SnO_2_-Sb Nanorods Anode for Electrochemical Degradation of CI Acid Red 73. J. Electrochem. Soc..

[34] Deng S., Dai Y., Situ Y., Liu D., Huang H. (2021). Preparation of nanosheet-based spherical Ti/SnO_2_-Sb electrode by in-situ hydrothermal method and its performance in the degradation of methylene blue. Electrochim. Acta.

[35] Chen Z., Dong S., Wang M., Hu Z., Chen H., Han Y., Yuan D. (2023). Construction of 3D Hierarchical Co(3)O(4)@CoFe-LDH Heterostructures with Effective Interfacial Charge Redistribution for Rechargeable Liquid/Solid Zn-Air Batteries. Inorg Chem.

[36] Hu Z., Guo C., Wang P., Guo R., Liu X., Tian Y. (2022). Electrochemical degradation of methylene blue by Pb modified porous SnO_2_ anode. Chemosphere.

[37] Duan T., Chen Y., Wen Q., Duan Y. (2015). Novel Composition Graded Ti/Ru–Sb–SnO_2_ Electrode Synthesized by Selective Electrodeposition and Its Application for Electrocatalytic Decolorization of Dyes. J. Phys. Chem. C.

[38] Sun Y., Cheng S., Mao Z., Lin Z., Ren X., Yu Z. (2020). High electrochemical activity of a Ti/SnO_2_-Sb electrode electrodeposited using deep eutectic solvent. Chemosphere.

[39] Berenguer R., Quijada C., Morallon E. (2009). Electrochemical characterization of SnO_2_ electrodes doped with Ru and Pt. Electrochim. Acta.

[40] Zhang H., Qian J., Zhang J., Xu J. (2022). A comparison study of TiO_2_@ATO@MOx (TAM, M = Mn, Fe, Co, Ni, Cu, and Zn) electrodes on the electrochemical activity and stability. J. Alloy. Compd..

[41] D’Angelo D., Filice S., Scarangella A., Iannazzo D., Compagnini G., Scalese S. (2019). Bi_2_O_3_/Nexar polymer nanocomposite membranes for azo dyes removal by UV–vis or visible light irradiation. Catal. Today.

[42] Exner K.S., Anton J., Jacob T., Over H. (2014). Controlling selectivity in the chlorine evolution reaction over RuO(2)-based catalysts. Angew Chem. Int. Ed. Engl..

[43] Sun Y., Xia R., Zhang J., Xu J. (2022). Preparation of ferric oxide for efficient electrocatalytic oxidation of methylene blue. Inorg. Chem. Commun..

