# Supplementary material for: Robust Self-Supported SnO2-Mn2O3@CC Electrode for Efficient Electrochemical Degradation of Cationic Blue X-GRRL Dye
Source: Molecules. 2023 May 8;28(9):3957. doi: 10.3390/molecules28093957 (PMC10180115; doi:10.3390/molecules28093957)
Supplement: Supplementary file 1 [file molecules-28-03957-s001.zip › molecules-2342658-supplementary.pdf]

Robust Self-Supported SnO_2-Mn_2O_3@CC Electrode for Efficient Electrochemical Degradation of Cationic Blue X-GRRL Dye

Caiyun Li [1], Peng Yi [1], Junwei Sun [2], Xi-Ao Wang [2], Rongzhan Liu [1,3,*], Jiankun Sun [2,3,*]

1 College of Textiles and Clothing, Qingdao University, Qingdao 266071, China

2 College of Chemistry and Chemical Engineering, Qingdao University, Qingdao 266071, China

3 Collaborative Innovation Center for Eco-Textiles of Shandong Province and the Ministry of Education, 308 Ningxia Road, Qingdao 266071, China

* Correspondence: lrz727602@qdu.edu.cn (R.L.); sunjk@qdu.edu.cn (J.S.)

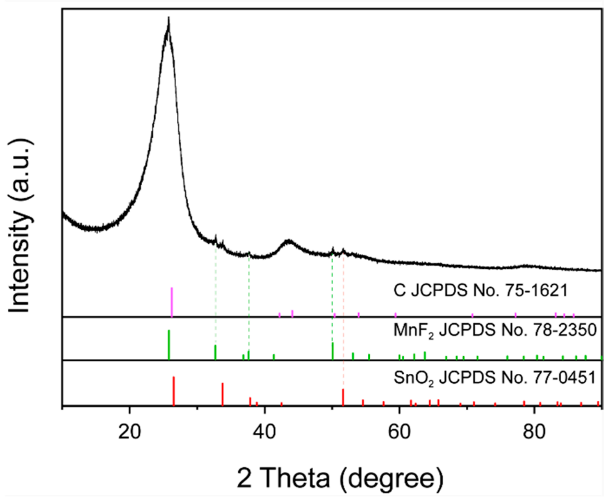

Figure S1. XRD pattern of SnO_2-Mn_2O_3@CC precursor.

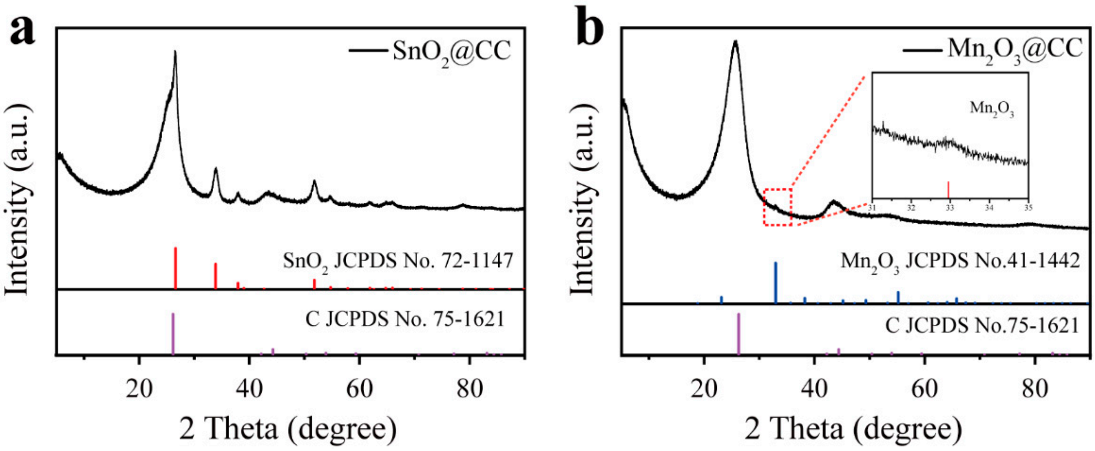

Figure S2. XRD patterns of SnO_2@CC (a) and Mn_2O_3@CC (b).

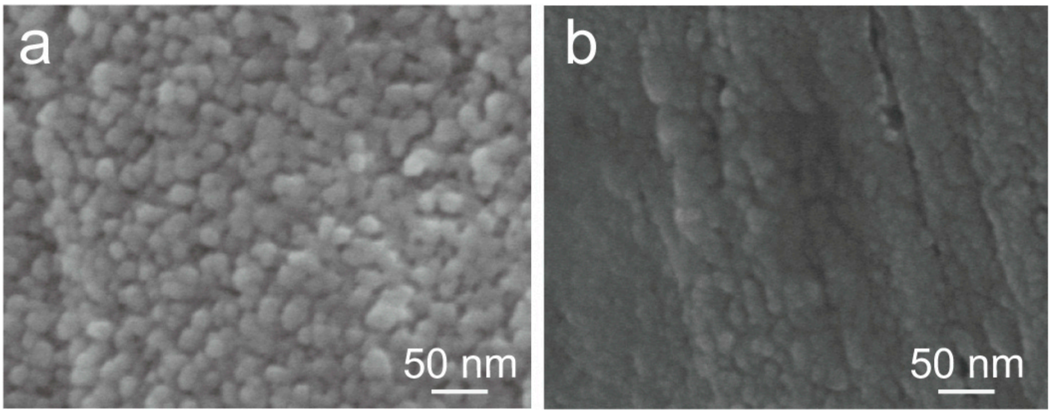

Figure S3 SEM images of Mn_2O_3@CC (a) and SnO_2@CC (b).

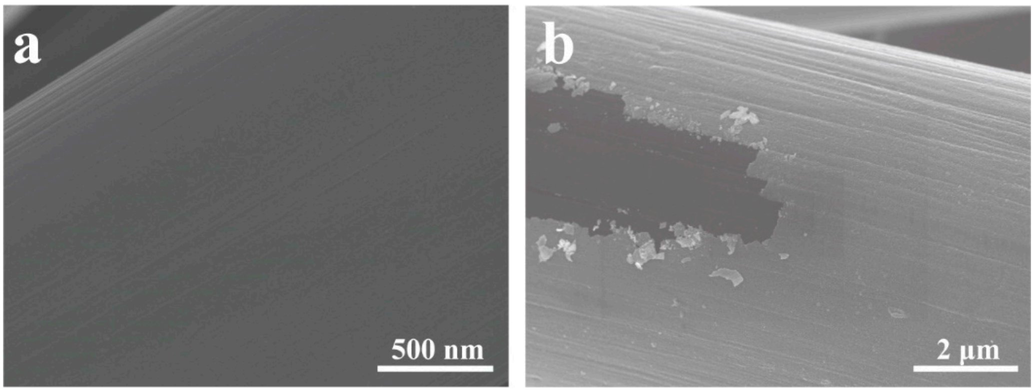

Figure S4 SEM images of carbon cloth (a) and SnO_2@CC (b).

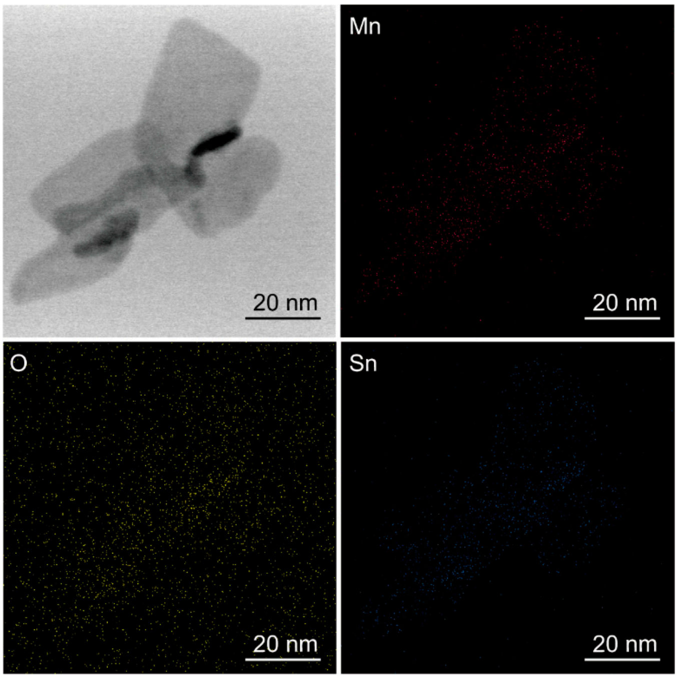

Figure S5 STEM image and corresponding EDS elemental mapping images of Sn, O, Mn.

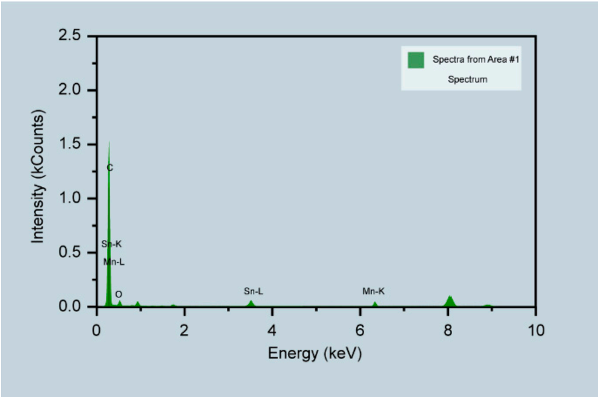

Figure S6. EDX spectrum of SnO_2-Mn_2O_3@CC.

Table S1 The elemental content in SnO_2-Mn_2O_3 hybrid nanosheets.

Element	Atomic Fraction (%)
O	66.67
Mn	0.19
Sn	0.53

Table S2 the electrical conductivity of different electrodes.

	Mn_2O_3@CC	SnO_2@CC	SnO_2-Mn_2O_3@CC
σ(S/m)*10^{-4}	5.13	6.67	9.52

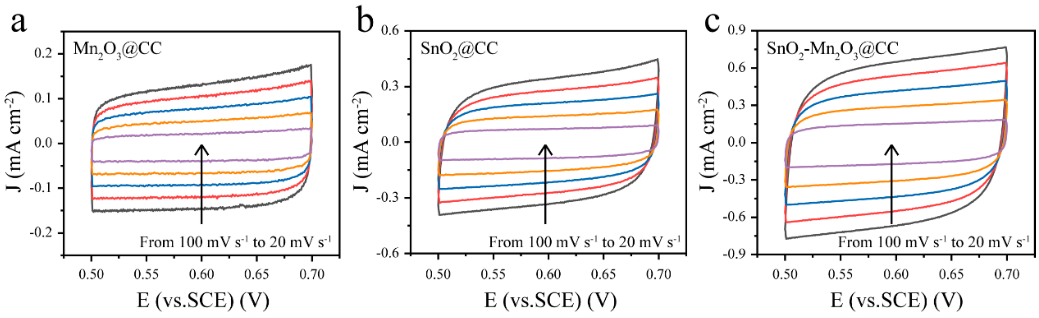

Figure S7. Cyclic voltammograms of Mn_2O_3@CC (a), SnO_2@CC (b), SnO_2-Mn_2O_3@CC (c) at the different scan rates varying from 20 to 100 mV s^{-1} in 1M KOH.

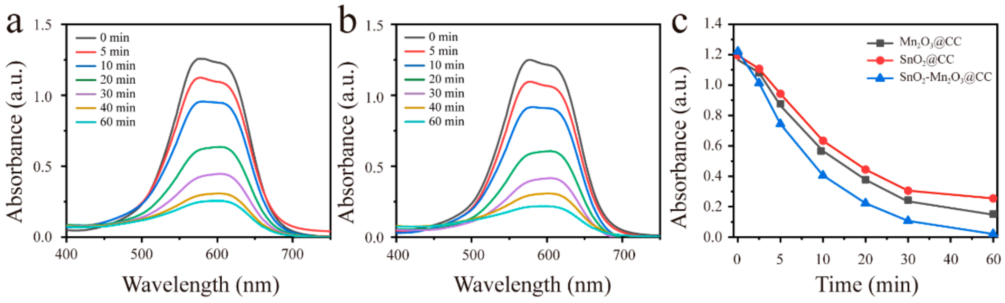

Figure S8. UV-vis absorbance spectrum of (a) Mn_2O_3@CC electrode and (b) SnO_2@CC electrode in the degradation process (initial concentration of cationic blue X-GRRL is 20 mg L^{-1}; supporting electrolyte is 0.5 M NaCl, current density is 15 mA cm^{-2}). (c) The comparative intensity of time-dependent characteristic adsorption peaks of cationic blue X-GRRL on

different electrodes.

Table S3 Kinetic parameters of different electrodes for degradation of cationic blue X-GRRL.

	K(min^{-1})	R^2
SnO_2-Mn_2O_3@CC	0.07096	99.6%
SnO_2@CC	0.04273	99.5%
Mn_2O_3@CC	0.04953	99.8%

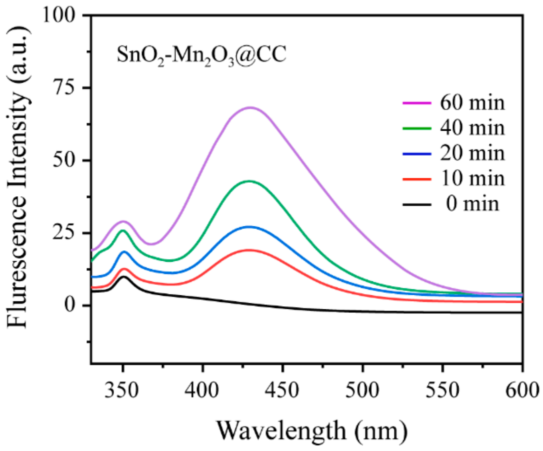

Figure S9. Variations of fluorescence intensity at different time (p-phthalic acid: 0.5 mM, NaCl: 0.5 M).

Table S4 Kinetic parameters of SnO_2-Mn_2O_3@CC electrode in different electrolytes for degradation of cationic blue X-GRRL.

	K(min^{-1})	R^2
0.5 M NaCl	0.07096	99.6
0.5 M Na_2SO_4	0.00373	99.3
0.5 M Na_2CO_3	0.03081	98.7
